# Metal-Based Nanomaterials: Work as Drugs and Carriers against Viral Infections

**DOI:** 10.3390/nano11082129

**Published:** 2021-08-20

**Authors:** Junlei Yang, Lihuan Yue, Zhu Yang, Yuqing Miao, Ruizhuo Ouyang, Yihong Hu

**Affiliations:** 1 Institute of Bismuth Science, University of Shanghai for Science and Technology, Shanghai 200093, China; fuyangjunlei@163.com (J.Y.); 203612304@st.usst.edu.cn (Z.Y.); yqmiao@usst.edu.cn (Y.M.); 2 Institut Pasteur of Shanghai, Chinese Academy of Sciences, University of Chinese Academy of Sciences, Yueyang Road 320, Shanghai 200031, China; 3 CAS Key Laboratory of Molecular Virology & Immunology, Institutional Center for Shared Technologies and Facilities, Pathogen Discovery and Big Data Center, Institut Pasteur of Shanghai, Chinese Academy of Sciences, Yueyang Road 320, Shanghai 200031, China; lhyue@ips.ac.cn; 4 Department of Bioengineering, School of Life Sciences and Biotechnology, Shanghai Jiao Tong University, Shanghai 200240, China

**Keywords:** metal-based nanomaterials, characteristics, antiviral therapy, mechanism, application

## Abstract

Virus infection is one of the threats to the health of organisms, and finding suitable antiviral agents is one of the main tasks of current researchers. Metal ions participate in multiple key reaction stages of organisms and maintain the important homeostasis of organisms. The application of synthetic metal-based nanomaterials as an antiviral therapy is a promising new research direction. Based on the application of synthetic metal-based nanomaterials in antiviral therapy, we summarize the research progress of metal-based nanomaterials in recent years. This review analyzes the three inhibition pathways of metal nanomaterials as antiviral therapeutic materials against viral infections, including direct inactivation, inhibition of virus adsorption and entry, and intracellular virus suppression; it further classifies and summarizes them according to their inhibition mechanisms. In addition, the use of metal nanomaterials as antiviral drug carriers and vaccine adjuvants is summarized. The analysis clarifies the antiviral mechanism of metal nanomaterials and broadens the application in the field of antiviral therapy.

## 1. Introduction

Virus infection has always been a threat to human and animal health. Typical viruses include hepatitis B virus [1], influenza virus [2], human immunodeficiency virus (HIV) [3], and coronavirus [4], etc., which can cause severe disease. Therefore, antiviral drug development is a major research direction for scientists. At present, the main treatment methods for viral infections include developing vaccines and screening antiviral drugs. However, the cycle for virus vaccine development and drug screening is currently too long. Additionally, the emergence of antiviral drug resistance has brought considerable challenges to successfully suppressing viral infections, so the development of new antiviral drugs is particularly important.

**Figure 1:**
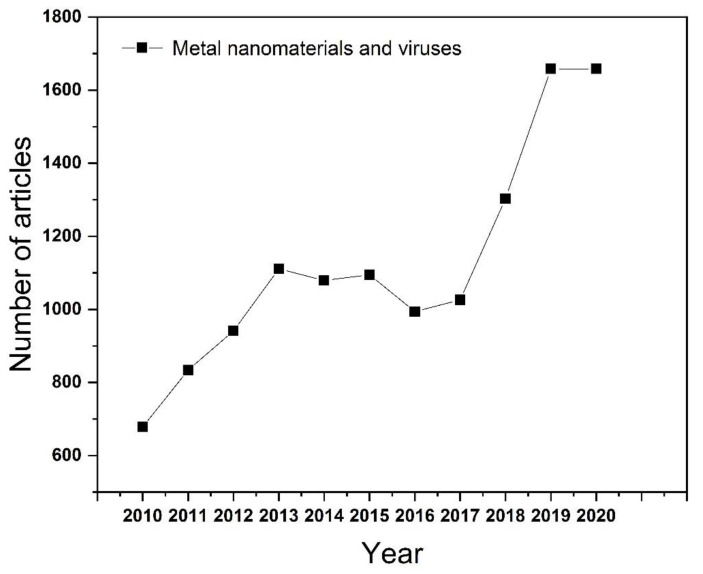
The number of articles published each year on the application of metallic nanomaterials in the field of viruses.

As one of the emerging fields in recent years, nanomaterials can be simply divided into one-dimensional, two-dimensional, and three-dimensional nanostructures according to the morphology of the material. There are many methods to synthesize nanomaterials, such as mechanical grinding synthesis [5], chemical vapor synthesis [6], chemical liquid reaction [7], physical vapor deposition [8], and phytosynthesis [9,10]. Among them, the phytosynthesis method is the most remarkable method of preparing nanoparticles now. It has the advantages of environmental protection and low energy consumption, but it also has the disadvantages of limited reaction yield and poor particle uniformity. Since then, further research has made substantial progress on the preparation methods of nanomaterials by adjusting the ratio of raw materials and exploring suitable reaction conditions. Nanomaterials have the advantages of small size, high specific surface area, adjustable particle size, and easier surface functionalization, which make them widely used in sensing [11], catalysis [12], energy storage [13], and the medical treatment field [14]. Metal-based nanomaterials could be widely used in biomedical fields, as metal ions are essential in living organisms. Since the particle size of the virus particles ranges from tens to hundreds of nanometers, the surface activity of the metal material is enhanced after the metal is nanosized. It can be used in the inhibition of virus infection. In addition, according to the statistics on Web of Science, the number of articles published on the application of metal nanomaterials in the field of viruses has been increasing year-on-year (Figure 1). More importantly, some nanomaterials such as Ag [15], Au [16], ZnO [17], etc., which have an inhibitive effect on bacteria and viruses, highlight their potential for antiviral applications. Notably, the pandemic of severe acute respiratory syndrome coronavirus 2 (SARS-CoV-2) in 2019 poses a huge threat to human health. As such, progress on virus pathogenesis new antiviral drug development is a research priority [18].

This review first provides a more comprehensive summary of the different stages of the role of metal nanomaterials in inhibiting viral infections in recent years. (1) Directly inactivating the virus; (2) inhibiting virus adsorption and entry; (3) intracellular virus suppression. Subsequently, it summarizes the use of metal-based nanomaterials as drugs and vaccine carriers. The application of metal nanomaterials in the field of antiviral research is introduced and its prospects are discussed. This review focuses on metal nanomaterials by comparing research progress in related fields in recent decades [19,20,21]. The inhibitory effect of metal nanomaterials on virus infection and their application as drugs and vaccine carriers are discussed and demonstrated.

## 2. Inhibition of Virus Infections

**Figure 2:**
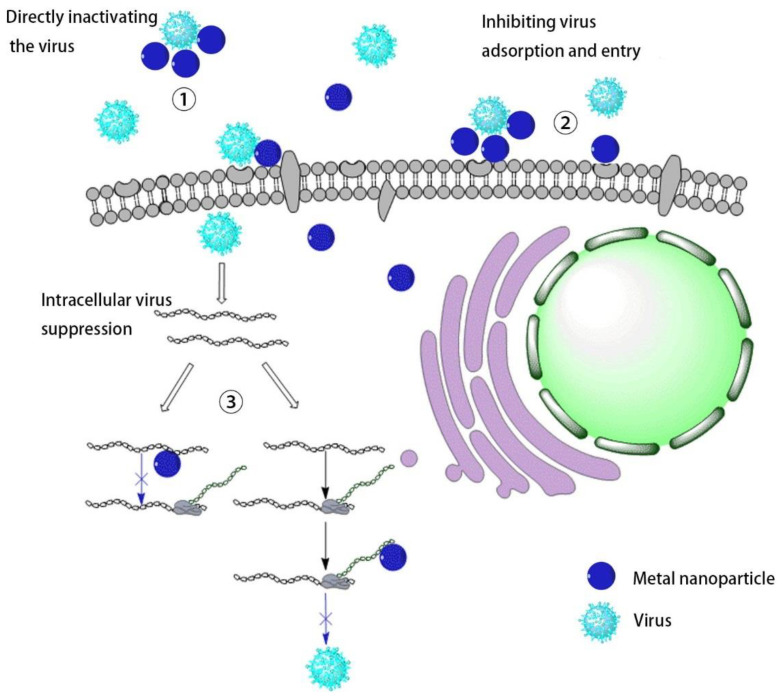
The mechanism of metallic nanomaterials inhibiting virus infection.

Virus infection of cells can be divided into three stages. The first is the early adsorption of the virus onto cell membrane, where the virus binds to cell surface receptors through its surface protein. The second is the process of uncoating, replication, and translation after the virus enters the cell. Finally, the virus repackages to release new progeny viruses. In these processes, if metal nanomaterials can inhibit the virus in the early stage and prevent the virus from invading cells, they can be used in medical protective equipment, which has great potential for preventing the virus spread (Figure 2).

### 2.1. Direct Inactivation of the Virus

**Figure 3:**
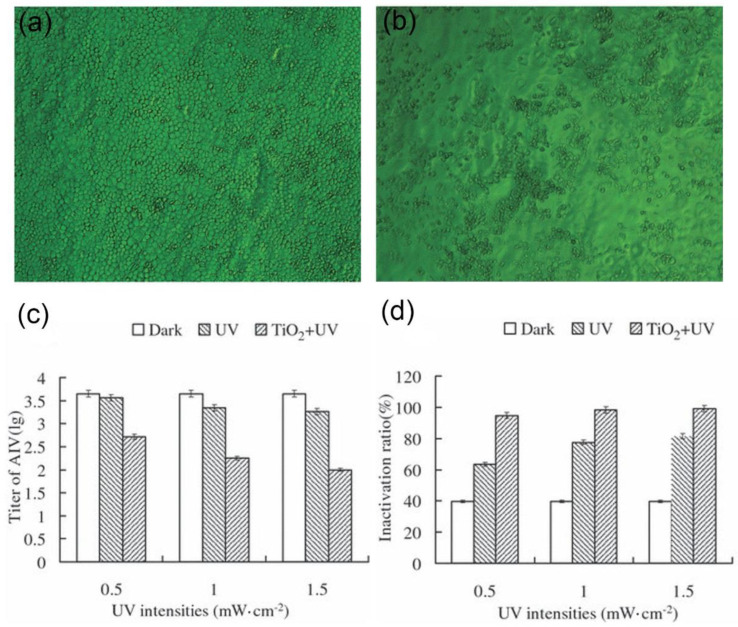
Cytopathic effect on Madin-Darby canine kidney cells infected with avian influenza virus eluted from nano-TiO_2_ film (**a**) and control (**b**); changes in titer (**c**) and inactivation efficiency (**d**) of H9N2 vs. ultraviolet intensity. (Reproduced with permission from Ref. [25], Copyright 2010, Wiley).

If metal-based nanomaterials inactivate viruses directly before viruses invade cells, this requires that nanomaterials possess excellent antiviral performance and good biological safety. Studies found that metal-based nanomaterials such as Au [22], Ag [23], ZnO [24], etc., have good antibacterial properties. Among them, TiO_2_ nanomaterials with photocatalytic properties can generate active substances by using light or through their affinity with biological macromolecules. They can damage the biofilm and kill the bacteria. Since the surface of the virus also has a biofilm, these metal-based nanomaterials can be used for the direct inactivation of the virus. H. Cui and colleagues found that TiO_2_ has good photocatalytic properties and can generate reactive oxygen species (ROS) under ultraviolet irradiation. ROS has oxidative properties. Therefore, using external interference in tumor therapy, the balance of ROS expression in tumor cells will be affected, which accelerates tumor cell apoptosis. At the same time, its excessive presence can also destroy the biofilm and cause it to lose its biological activity. Related genes in cells regulate the normal expression level of ROS. H. Cui’s group explored the inhibitory effect of TiO_2_ on the influenza virus under ultraviolet irradiation (Figure 3). Compared with normal cells infected with influenza virus (Figure 3b), the cell activity after TiO_2_ treatment is greatly enhanced (Figure 3a), which intuitively shows the inhibitory effect of TiO_2_ on the virus. Later studies showed that with the increase of TiO_2_, virus culture time, and ultraviolet light time, the inhibitory effect of TiO_2_ on the virus is increased. Considering that the material can generate ROS under ultraviolet light irradiation, it can inactivate viruses [25]. Similarly, N. A. Mazurkova explored the antiviral properties of TiO_2_ under sunlight and ultraviolet rays. It was observed by scanning electron microscopy that the material adsorbed to the surface of the virus envelope in the early stage, and then destroyed it, thereby inactivating the virus. Later experimental results showed that the inhibitory effect was positively correlated with the concentration of TiO_2_ and the incubation time [26]. Ahmad Tavakoli and colleagues also used CuO nanomaterials to generate ROS and explored its inhibitory effect on herpes simplex virus (HSV). During the study, CuO was added after the virus infected the cells. After 48 h of culture, the results showed that the inhibitory effect increased as the CuO concentration increased [27].

Compared with some metal-based nanomaterials that use light to produce ROS to inactivate viruses, the mutual combination of metal ions and protein molecules can often change the protein conformation, causing irreversible damage to the effect of inhibiting virus infection. SungJun Park synthesized a magnetic hybrid colloid loaded with Ag nanoparticles of different sizes. Using the interaction between Ag and biological macromolecules, this nanomaterial’s inhibitory effect on bacteriophages, norovirus, and adenovirus was explored. The results show that the virus binds to the sulfhydryl-containing protein on the surface of the virus through Ag nanoparticles, thereby destroying the virus envelope to inhibit the virus. Similarly, the use of the affinity of metal ions with proteins can bind to the outer surface proteins of virus particles and destroy the virus structure to inhibit the virus [28]. F. Pfaff tested its inhibitory effect on modified vaccinia virus Ankara (MVA), human adenovirus serotype 5 (HAdV-5), poliovirus type 1 (PV-1), and murine norovirus (MNV) by co-cultivating WC material and virus. The author shows that WC tends to reunite, which can encapsulate virus particles, thereby destroying the nucleic acid of the virus and inactivating the virus [29].

### 2.2. Inhibiting Virus Adsorption and Entry

The binding process of the virus and the cell is through the targeted binding of the envelope protein on the virus surface to the cell surface receptor. The expression of these virus surface proteins is stable and not prone to gene mutation. Therefore, nanomaterials can be used to adsorb viruses directly, simulate virus-connected receptors on the cell surface, and competitively bind virus particles before the virus invades the cell to prevent the virus from infecting the cell. The mechanism provides a new trend for the research of antiviral materials.

**Figure 4:**
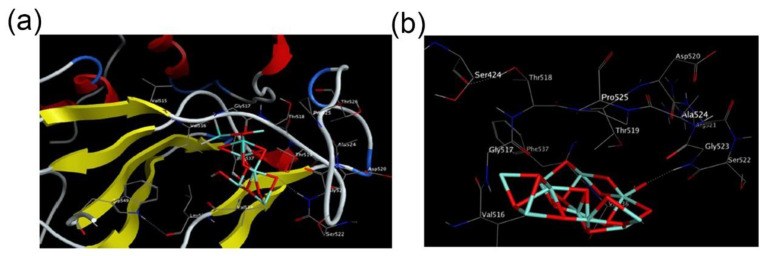
Three-dimensional interaction diagram showing (**a**) Fe_2_O_3_; and (**b**) Fe_3_O_4_ docking interactions with the key amino acids in the HCV E2 glycoprotein. Three-dimensional interaction diagram showing docking interactions with the key amino acids in the HCV E2 glycoprotein. (Reproduced with permission from Ref. [31], Copyright 2020, Elsevier).

To prevent the virus from invading the cell, scientists target the combination of virus and cell. The most direct method can be used to bind the viral capsid protein to occupy the receptor of the virus-bound cell, thereby preventing the disease from invading the cell. Metal ions have a good affinity with biological macromolecules, and the combination of virus and metal nanomaterials inhibits virus infection on cells. Rishikesh Kumar’s lab has synthesized 10 nm Fe_2_O_3_ nanoparticles with the external glycine reducing the biological toxicity of Fe_2_O_3_ nanomaterials, and this shows a specific inhibitory effect on influenza virus H1N1. The results show that, with smaller particle size and a higher specific surface area, the combination of Fe_2_O_3_ nanomaterials and viruses is easier. The Fe_2_O_3_ nanoparticles combine with viruses to produce a particular steric hindrance, inhibiting the virus from invading cells [30]. Yasmin Abo-Zeid explored the interaction of Fe_2_O_3_/Fe_3_O_4_ with hepatitis C glycoproteins E1, E2, and the spike protein receptor binding domain of COVID-19, respectively. She found that the ends of these viral proteins have richer amino acid residues. However, iron oxide nanoparticles can bind to them to occupy the virus’s binding site and the cell, successfully interfering with the absorption of the virus into the cell (Figure 4) [31].

**Figure 5:**
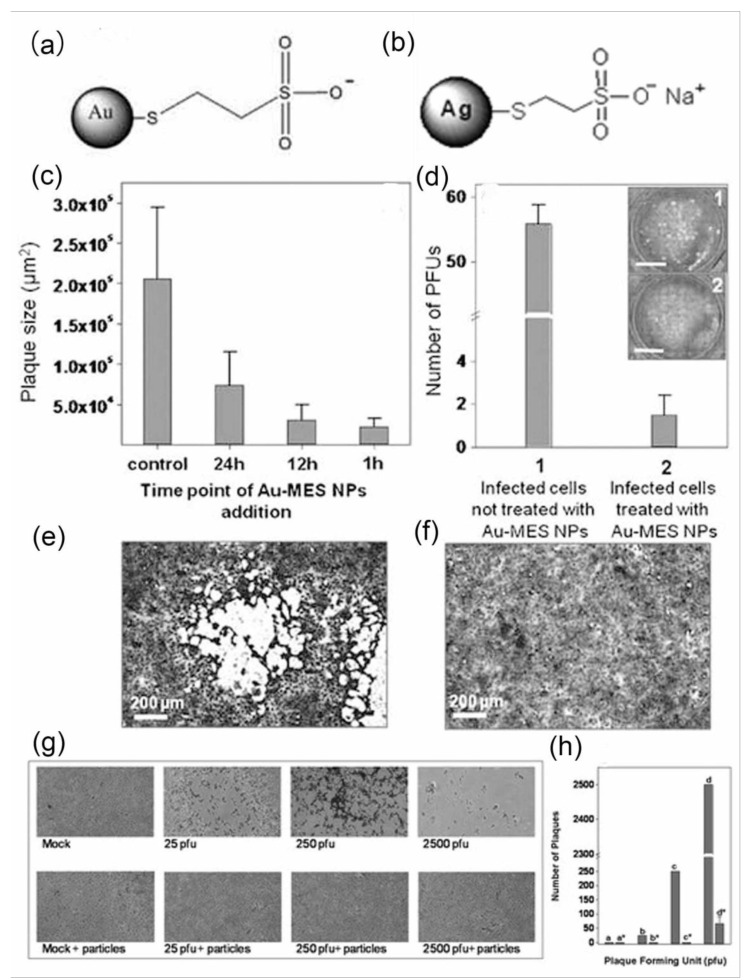
Characterization of (**a**) Au-MES (MES = mercaptoethane sulfonate) NPs; (**b**) Ag-MES NPs; (**c**) variation of plaque size as a function of increasing time lapse between primary infection and the administration of Au-MES NPs; (**d**) evaluation of inhibition of virus entry into host cells. Plaque numbers observed 48 h after infection (1) without and (2) with nanoparticles; infected cell culture in the absence of Au-MES NPs (**e**) and in the presence of Au-MES NPs (**f**). (Reproduced by permission from Ref. [22], Copyright 2010, Wiley.) (**g**) Optical microscopic image (×10 magnification) demonstrating the effect of Ag-MES nanoparticles on HSV-1 infectivity; (**h**) quantitative analysis of the Ag-MES inhibition efficiency (reproduced with permission from Ref. [23], Copyright 2009, American Chemical Society).

**Figure 6:**
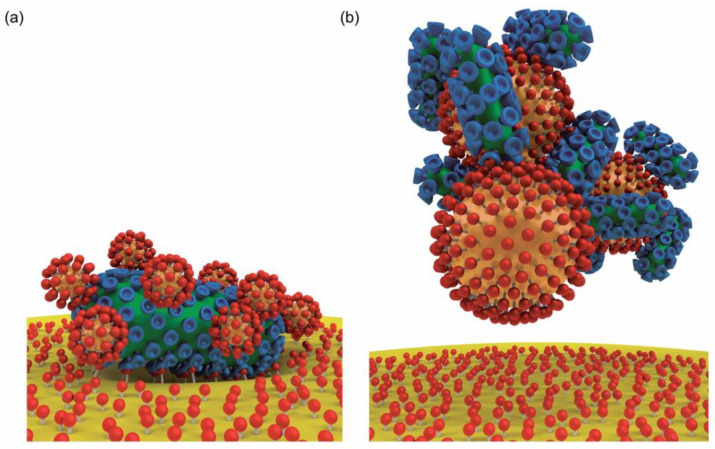
Schematic representation of the size-dependent virus inhibition by ligand functionalized gold nanoparticles according to the TEM data. (**a**) Although smaller sized gold nanoparticles decorate virions, the inhibition of virus-cell binding was shown to be inefficient; (**b**) larger virus-sized gold nanoparticles induce the formation of virus-inhibitor clusters, inhibiting the virus-cell binding more efficiently. (Reproduced with permission from Ref. [33], Copyright 2014, Royal Society of Chemistry).

Metal-based nanomaterials have a small particle size and a high specific surface area, convenient for material modification, and can be functionalized in combination with the characteristics of virus receptors on cell surface. Studies have shown that when HSV invades cells, glycoproteins on the envelope’s surface bind to heparin sulfate (HS) on the cell membrane [32]. In this binding process, the sulfonate ion in the protein plays an important role. Therefore, the surface of metal-based nanomaterials can be functionalized to modify sulfonate groups and simulate HS on the cells’ outer surface to prevent viruses from invading cells through competitive binding with viruses. Dana Baram-Pinto and colleagues used the affinity of the noble metals (Au and Ag) to sulfhydryl groups and external sulfonate ions as shown in Figure 5 to synthesize metal-based nanomaterials that simulate HS. Studies have shown that the material has successfully added sulfhydryl sulfonate, and the later anti-HSV experiments have shown that it has good virus inhibition characteristics. Influenza virus invades cells through the highly conserved protein hemagglutinin (HA), which contains six disulfide bonds on the surface of the virus, binding cell surface receptors and entering the cell. The study of metal nanoparticles focuses on binding HA and destroying its structure to prevent viruses from invading cells [22,23]. In addition, Jonathan Vonnemann explored the strength of the adsorption between Au and viruses by synthesizing Au with different particle sizes and then connecting sulfonate particles. The results show that when the diameter of Au nanoparticles is less than 52 nm, although the nanomaterials are adsorbed on the virus envelope, the Au nanoparticles’ surface area is not enough to prevent the adsorption of the virus and cells, as shown in Figure 6. When Au nanoparticles are larger than 52 nm, the nanoparticles occupy the surface area of the virus. The steric hindrance is increased, which significantly reduces the contact between the virus and the cell, thereby reducing the probability of the virus infecting cells [33].

Au/Ag is known to have a good affinity with S. Jinyoung Kim synthesized Au and Ag with different morphologies and with or without holes. After co-cultivating with the virus for a certain period, the adsorbed and unadsorbed viruses were separated by centrifugation. The interaction between the nanoparticles and the virus was observed by transmission electron microscope, and the number of disulfide bonds in the viral hemagglutinin was quantified to determine the effective destruction of the disulfide bonds by the metal-based nanoparticles. The results show that, compared with non-porous Au and Ag nanomaterials, porous Au nanoparticles can achieve a more apparent antiviral effect by destroying the disulfide bonds in the hemagglutinin on the surface of the virus [34]. This provides a new application direction for porous Au nanoparticles to suppress viruses containing enveloped spike proteins.

The process of a virus invading cells involves specific binding, which provides a good starting point for preventing viruses from infecting cells. The primary inhibition method is to competitively bind to the virus by simulating cell surface virus receptors and inhibiting the virus from invading cells. However, this method can stop the virus from spreading further and does not inactivate the virus. There are certain drawbacks to the application. It is worth noting that these nanomaterials can be adsorbed on medical protective fabrics and concrete surfaces, thereby reducing the spread and infection of viruses.

### 2.3. Intracellular Virus Suppression

After the virus enters the cell, it uses the intracellular machinery to carry out protein replication and translation. Since the virus is uncoated to expose the genetic material DNA/RNA, this process can provide the possibility for external drugs to destroy the viral nucleic acid or interfere with the process of translation, replication, and release.

When the virus enters the cell, it begins the process of unpacking, replication, and translation. During this period, the RNA/DNA and protein of the virus are synthesized in large quantities; this happens to be the most effective moment for drugs to inactivate viruses. By importing the drug into the cell, it acts on the viral RNA/DNA and protein, destroying the replication process of the virus and inhibiting its reproduction. Most nanomaterials have good biocompatibility. The advantages of small particle size and high specific surface area can help nanomaterials enter cells efficiently. With the capability of binding metal ions with biological macromolecules, the metal nanomaterials electrostatically interact with viral nucleic acids or proteins to form chemical bonds, destroy their structures, or cause irreversible conformational changes of viral proteins, thereby achieving the purpose of inhibiting virus replication. Ting Du synthesized glutathione-encapsulated Ag_2_S nanoparticles to explore their inhibitory effect on coronaviruses. The author explored the expression levels of the virus by adding Ag_2_S materials at different times after virus infection. Since the particle size of Ag_2_S is about 2.5 nm, it has a more profound cell penetration ability and can better interact with viruses. PEDV nucleocapsid (N) protein is a protein that binds to viral RNA, and its expression is tested to verify the inhibition of Ag_2_S. The results show that Ag_2_S inhibits the virus by inhibiting the negative-strand RNA synthesis and budding of PEDV [35].

The relatively high surface activity of metal nanomaterials also brings the disadvantage of high cytotoxicity. However, more amphiphilic polymers such as PEG and PVP have been studied at this stage [36]. They can be easily combined with metal nanoparticles to enhance the water solubility of materials, reduce biological toxicity, and increase the internalization rate of cells to materials. ZnO is the main component of many sunscreens [37] and antibacterial materials [38], and its application in the field of antibacterial products has been extensively studied. However, the effect of ZnO on viruses is less reported. Studies have shown that synthetic micron-sized filamentous foot-like ZnO prevents HSV-1 from entering the cell [17]. Later studies showed that naked ZnO and PEG-modified ZnO had a dramatic inactivating effect on HSV-1, which was mainly achieved by interfering with the expression of early viral proteins. The results showed that PEG-modified ZnO had a better virus inhibition effect [39]. Similarly, through the inhibition of naked ZnO and PEG-modified ZnO on H1N1 influenza virus, the virus inhibition stage is shown to be after the virus entry [24].

## 3. Loading Drug Synergy

The high infectivity and pathogenicity of viruses has caused people to pay more attention to antiviral drug research and development. The current research and development of viral drugs mainly include screening natural drugs and synthesizing organic small-molecule drugs. However, the screening of natural medicines requires a high workload, and the inhibitory effect of natural medicines on viruses is not satisfactory. The inhibition mechanism remains to be explored. The research and development of organic small-molecule drugs, finding the relevant derivatives of existing antiviral small-molecule drugs, and further exploring drugs with viral inhibitory effects have good application prospects. However, small organic molecules have disadvantages such as low water solubility, poor biocompatibility, and high toxicity. Moreover, the development and application of vaccines have also opened up new areas for virus therapy. Vaccines are preventive treatments that can significantly reduce the incidence of viral infections. However, vaccines for injection have biological activity, and instability in transportation and storage is still an open question. Hence, many scholars have developed numerous carrier materials, such as colloids [40], magnetic nanomaterials [41], inorganic nanomaterials [42], and metal organic framework materials [43], to increase the loading rate of drug molecules by high biocompatibility and high specific surface area of the carrier material. Thereby, the carrier nanomaterial increases the uptake rate of the cell to the drug, and enhances the stability of the vaccine and the immune response of the body.

The human body contains many kinds of metal elements, including Al, Ca, Mg, Fe, Na, Mn, Zn, K, Li, Cu, Se, etc. These metals are essential for participating in various life cycle processes in the human body. Therefore, small amounts of metal ions are less harmful to the human body, and the metal ions could be prepared as nanomaterials with a high specific surface area of nanomaterials, porosity of some materials, and good biological activity as a promising drug carrier.

### 3.1. Loading Drugs

The poor water solubility of antiviral drugs makes their bioavailability low, and higher doses are often required to achieve the desired therapeutic effect. However, higher doses will produce certain toxicity to organisms, so the emergence of drug carriers can improve the drugs’ bioavailability and reduce the damage to organisms. When selecting metal nanoparticles as drug carriers, the toxicity of metal ions to organisms and the loading efficiency of materials should be considered. Using metal ions or inert ions, with higher content in human cells, can reduce the toxicity of materials and increase the materials’ natural metabolism.

Human Acquired Immune Deficiency Syndrome (AIDS), caused by HIV, is a disease in which immune cells are the primary targets. These cells can accumulate viruses for a long time to be used as targeted sites for drug delivery. Studies have shown that Au nanoparticles can be more internalized by human macrophages and have less biological toxicity [44]. Hinojal Zazo and colleagues immobilized stavudine, an antiretroviral drug, on the surface of Au nanoparticles to deliver targeted drugs to macrophages. In addition, when Au is loaded with drugs, it can increase the expression of inflammatory genes in macrophages and induce a specific inflammatory response in the body [45].

Quantum dots (QD) are a type of low-dimensional semiconductor material and often have smaller sizes. Therefore, applying QD materials to the field of biological therapy can often improve the therapeutic effect. Ranjeet Dungdung’s group used ZnS quantum dots as a drug carrier, loaded with mycophenolic acid (MPA), an immunosuppressant against dengue fever virus. It was found that cells have a higher internalization rate of ZnS-coupled MPA, and its inhibitory effect on dengue virus was significantly improved and the selectivity index was increased by two orders of magnitude [46]. The study shows that quantum dots can significantly increase the uptake rate and therapeutic effect of drugs, indicating that QD as drug carriers have great application prospects.

**Figure 7:**
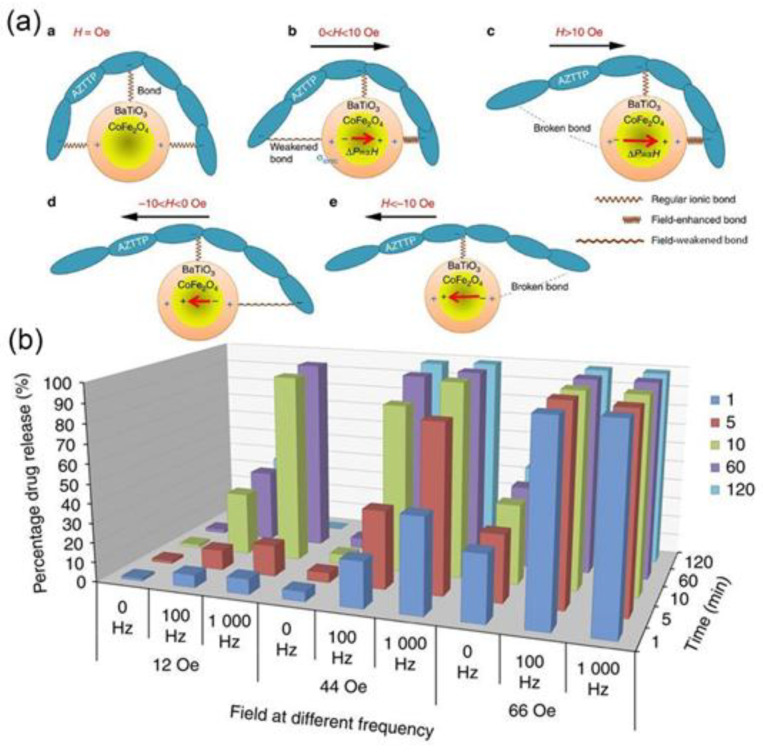
(**a**) Illustration of the underlying physics of the a.c.-field-triggered release; (**b**) pharmacokinetics study: three-dimensional chart representation of the drug release percentage at various combinations of the field strength, the frequency, and the treatment duration. (Reproduced with permission from Ref. [47], Copyright 2013, *Nature* Publishing Group).

The application of antiviral drugs can significantly reduce the infection rate of the virus. However, the presence of the blood–brain barrier reduces the scope of action of the drug, which is also a shortcoming in the therapeutic application of most drugs. When Madhavan Nair and colleagues studied the antiretroviral application of HIV, they found that the antiretroviral drug azidothymidine 5′-triphosphate (AZTTP) has low efficiency in passing through the blood–brain barrier, which makes the treatment of the virus not very efficient. Therefore, it is crucial to find an excellent carrier to load AZTTP to achieve targeted drug release. Studies have shown that by synthesizing 30 nm magnetic CoFe_2_O_4_@BaTiO_3_, AZTTP is bonded to nanomaterials’ surface through ionic bonds. As shown in Figure 7, the delivery and trigger release of the drug are controlled by applying the strength and frequency of the magnetic field in different directions to achieve the purpose of release on demand. Later studies have shown that the load rate of AZTTP for three hours at 37 °C is about 24% through spectrophotometric detection. Depending on the magnetic field strength and frequency, they are adjusted to achieve quantitative release of AZTTP [47].

**Figure 8:**
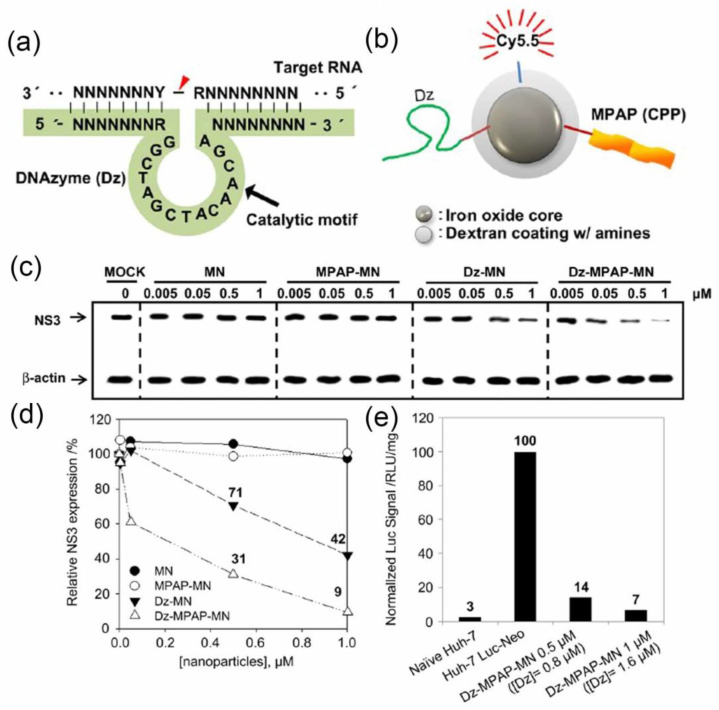
Material construction and performance testing. (**a**) Structure of the 10–23 synthetic DNAzyme (shaded in green color). A conserved 15-base unpaired motif serves as the catalytic core and is flanked by variable binding domains at its 50- and 30-ends. The point of scission within target RNA is indicated by a red color arrow; (**b**) multifunctional nanoparticle formulation for DNAzyme delivery. (Dz, DNAzyme; MPAP, myristoylated polyarginine peptide; Cy5.5, fluorescent dye; CPP, cell-penetrating peptide); (**c**) Western blot analysis of HCV NS3 expression in cultured Huh-7 Luc-Neo cells treated with Dz-conjugated multifunctional nanoparticles; (**d**) densitometric analysis of the data shown in (**c**). Density values for NS3 were normalized to the β-actin band density; (**e**) luciferase (reporter gene) assays of Huh-7 Luc-Neo cells treated with Dz-MPAP-MN indicate dose-dependent downregulation of the NS3 target gene. (Reproduced with permission from Ref. [51], Copyright 2012, Elsevier).

In the treatment of viral infections, in addition to traditional antiviral drugs, nucleic acid drugs such as oligonucleotides [48], siDNA [49], DNAzymes [50], etc., have also achieved good results. These nucleic acid biological macromolecules can often target the RNA fragments of the virus and interfere with the virus’s replication process in the cell to achieve the purpose of inhibition. However, these nucleic acid drugs still have the disadvantages of low cell uptake rate and low bioavailability. Using these nucleic acid drugs, the methods of transporting them into the cell through the carrier can increase the uptake rate of nucleic acid drugs to the cell and improve the therapeutic effect. Soo-Ryoon Ryoo and colleagues selected a therapeutic oligonucleotide called DNAzyme that specifically targets hepatitis C virus (HCV) mRNA and splits specific genes N53, a helicase and ribozyme gene involved in hepatitis C virus replication. As shown in Figure 8a,b, iron oxide nanoparticles (MN) were conjugated to DNAzyme as a carrier, and then connected to the outer layer of cell-penetrating peptide (CPP) through disulfide bonds and modified fluorescein CyC5.5 (D2-MPAP-MN). The material group connected with DNAzyme showed a significant inhibitory effect (Figure 8c–e), by examining the gene expression of virus N53, compared with the carrier material (MN/MPAP-MN) [51].

Studies have shown that small interfering RNA 331 (siRNA331) can target the conserved region of hepatitis C virus RNA [50]. Inspired by this, Zhongliang Wang and colleagues used Au nanoparticles as a carrier platform to link endoribonucleotides and DNA oligonucleotides on the outer surface through non-covalent adsorption and Au-S bonds to form nanozymes. Here, endoribonucleotides are used for non-specific degradation of single-stranded RNA. The DNA oligonucleotide design contains siRNA331, which is used to improve the hybridization and cleavage efficiency of the targeted gene of nanozyme. The nanozyme specifically binds HCV RNA and cuts it to inhibit the replication process of HCV. Through specific experiments in the later period, it was found that nanozymes have a better ability to precisely cut HCV genes. The experimental results in cells and mice are consistent with expected results [52].

### 3.2. Load Vaccine

Vaccines are used to prevent infectious diseases by artificially attenuating and inactivating pathogenic microorganisms and their metabolites to produce an immune response in the body. After immunization, the organism will produce immune antibodies to protect itself, and the immune system will form a memory of pathogenic microorganisms. When it invades again, the immune system can react in time and produce corresponding antibodies to protect the organism. The treatment of viral infections is often more complicated. However, it is often a valid preventive method to cause the body to produce specific antibodies through the previous vaccine injection.

Vaccines can be divided into live-attenuated vaccines, inactivated vaccines, recombinant vaccines, and so on. Among them, live vaccines (adenovirus vaccines [53], measles vaccines [54], and polio vaccines [55]) can stimulate the body’s comprehensive systemic immunity and long-lasting immune response with the disadvantages of antigen interference and enhanced virulence. Inactivated vaccine (influenza split vaccine [56], rabies vaccine [57], and hepatitis A vaccine [58]) are viruses that have been inactivated by chemical or physical methods, and still maintain the immunogenicity of their immune antigens. Killed vaccines have high safety and good stability. However, adjuvants are often needed to enhance the immune effect. Gene vaccines (hepatitis B vaccine [59], HIV vaccine [60]) are not infectious, convenient for mass production, and safe. They also need adjuvants to enhance the immune effect during usage. Therefore, adding vaccine adjuvants to non-specifically increase the body’s immune response in traditional vaccine production is often a good preparation plan. These adjuvants often increase the immune response ability in organisms through their own physical and chemical properties or by changing antigens’ physical properties.

For nanoparticles, by relying on the diversity of their structures and morphologies, they can be used as adjuvants and suitable carriers of antigens to enhance the immune response. This requires easy functional modification of nanomaterials’ surface, and the vaccine adjuvants are physically or chemically connected. So, the adjuvant is released slowly to achieve the effect of prolonging the immune time. Meanwhile, some metal-based nanomaterials have adjuvant properties, and their use with vaccines can increase the body’s immune effect. Besides, the affinity of metal ions and biological macromolecules can be used to combine the surface proteins of the virus in the vaccine to enhance its stability, thereby improving the immune effect.

On the other hand, the storage and transportation of vaccines are more challenging, and the biological activity needs to be guaranteed. The vaccine can be protected by adding nanomaterials to increase its stability during transportation and storage and reduce costs. They are widely used as carriers in the biological field [61]. Metal-organic framework materials are nanomaterials with porous structures. Their surface is rich in unsaturated metal sites and is easier to modify after synthesis. Therefore, it is widely used in drug transportation [62]. In the same way, vaccine reagents are biologically active and can use porous materials to be adsorbed in the pores through static electricity to achieve the effect of vaccine protection. In addition, the affinity of metal ions and biological proteins is used to coordinate and adsorb the metal nanomaterials and vaccine proteins to ensure the biological activity of the vaccine. This indicates extensive application prospects in the field of vaccine protection with metal nanomaterials.

ZIF-8 has mild synthesis conditions, and it is possible to encode ovalbumin (OVA), an antigen model, into ZIF-8 for co-transport of antigen and adjuvant by surface electrostatic adsorption of immune adjuvant cytosine-phosphate-guanine (CpG) [63]. According to the characteristics of ZIF-8 in which protonation in a weakly acidic environment leads to structural degradation, the release of antigens and adjuvants is achieved, and a robust immune response is caused. Similarly, Yong Yang [64] first synthesized MIL-101-Fe-NH_2_, which is safe and degradable, and then modified disulfide bonds on the outer surface. The immune adjuvant OVA is bound to the outer surface through disulfide bonds, and then CpG is adsorbed on the outer surface of MIL-101-Fe-NH_2_ through electrostatic adsorption. Since the glutathione (GSH) content in the cell is higher than that in the extracellular tissue, the synthetic material undergoes a redox reaction between GSH and the disulfide bond in the material to release OVA. Studies have shown that the material’s better transportation performance increases the immune effect and the memory of the immune system. Furthermore, metal elemental nanomaterials have a small particle size and are easily taken up by cells. Therefore, the cellular uptake of antigens and adjuvants that they carried increases [65,66]. More importantly, studies using Ag as the metallic element combined with influenza-inactivated virus vaccines have shown that the Ag nanoparticles can stimulate the regeneration of bronchial-associated lymphoid tissues and the expression of antigen-specific IgA, acting as an immune adjuvant [67]. Moreover, calcium phosphate nanoparticles (CAP) have been proven to be a good vaccine adjuvant [68,69].

Due to the excellent physical and chemical properties of metal-based nanoparticles, their biological applications have been continuously expanded. Previous work has found that some metal-based materials themselves have adjuvant properties. During the early vaccination process, alum is a promising adjuvant widely used in vaccines. Later studies found that CAP can stimulate the organism’s immune response, and its effect is better than that of alum [68]. On this basis, Marina A. Volkova and colleagues studied the immune response induced by CAP when combined with an inactivated Newcastle disease virus vaccine. It is found that CAP can induce effective mucosal immunity, thereby enhancing the effectiveness of the vaccine. CAP can also be used as a vaccine carrier. By synthesizing CAP nanoparticles, the immunologically active TLR9 ligand and influenza A antigen are loaded. Analogously, studies have shown that the loaded CAP nanoparticles can be effectively taken up by dendritic cells and can stimulate an effective immune response in the body [70].

Some adjuvants need to be added into inactivated virus vaccines to increase the immune effect when used in vivo. Here, adjuvants can stimulate the immune response, and protect the activity of the inactivated virus antigen. The inactivated foot-and-mouth-disease virus vaccine is not stable when used. The histidine residues on the surface protein are protonated, causing it to lose immune activity [71,72]. Metal ions have a good affinity with amino acid residues. Shuai Li and colleagues coordinated with histidine residues on the surface protein of the foot-and-mouth-disease virus through Zn^2+^ to prevent protonation in organisms and improve the stability of antigens [73]. Similarly, by taking advantage of the good affinity of metal ions and biological macromolecules, Ni coordination will bind to the virion and the target protein, activate the antigen-presenting cells in the body, and promote the efficiency of antigen cross-presentation [74]. In terms of vaccine transportation protection, Michael A. Luzuriaga selected the metal organic framework material ZIF-8 with milder synthesis conditions as the protection model. By carrying out surface biomineralization on the surface of the tobacco mosaic virus, the growth of the ZIF-8 shell achieves its purpose of protection under changing environmental conditions. After treatment with pressure, heating, methanol, guanidine hydrochloride, and ethyl acetate, compared to the protected virus, the degree of change in the surface protein of the virus after wrapping ZIF-8 is significantly smaller, which protects the virus from the external environment. After peeling off the outer layer of ZIF-8, it was found that the surface protein and RNA of the virus remained active. The subsequent results of mouse experiments also confirmed the above results [75].

We briefly summarize below the therapeutic mechanism of nanomaterials in viral infection inhibition. As shown in Table 1, according to different viral inhibition mechanisms, it is divided into direct inactivating, inhibition of absorption, intracellular inhibition, and as a carrier. The analysis shows that the metal nanometer that directly inactivates the virus mainly destroys the virus envelope by contacting the virus or releasing active substances to damage the virus. Inhibition of virus absorption is via competitive binding to cells, or binding the surface of the virus through metal nanomaterials, occupying the binding site of the virus and the cell to prevent the virus from invading the cell. Intracellular inhibition uses metal nanomaterials to enter the virus-infected cell, and metal ions damage the viral genetic material or interfere with its replication process to inhibit further virus replication. With high biocompatibility and high cellular uptake rate, the metal nanomaterial is used as a carrier to improve the therapeutic effect of antiviral drugs. In addition, metal nanomaterials are also used as vaccine adjuvants to improve immune response and enhance vaccine effects.

## 4. Conclusions

The spread of viruses such as SARS-CoV and SARS-CoV-2 as well as influenza viruses poses a huge threat to human health. It is generally known that viruses are prone to mutate due to external influences to produce different virus subtypes, limiting the use of traditional antiviral drugs and vaccines. Therefore, novel drug discovery and vaccine development for the treatment of viral infectious diseases is very important.

Metal-based nanomaterials have the advantages of high specific surface area and small particle size, making them widely used in the biological field. In terms of antiviral activity, some gold-based nanomaterials such as Au, Ag, CuI, TiO_2_, etc., have virus-inactivating ability and can damage their surface proteins or their genetic material by combining with viruses. In comparison, nanoparticles such as ZnO and Fe_3_O_4_ can prevent the virus from further infecting cells by interfering with the replication, translation, and release of the virus. Some other metal nanoparticles have a small particle size and good biocompatibility, are easily taken up by cells, and can be used as a suitable carrier for antiviral drugs. Moreover, utilizing the binding of metal ions and biological macromolecules, metal nanoparticles can be used as vaccine adjuvants or adjuvant carriers to promote the occurrence of immune response of the body. However, there are still some problems to be solved in the antiviral application of metal-based nanomaterials. First of all, the toxicity of metal ions in organisms is still a major obstacle to their application. The biological toxicity of metal ions has always been a major obstacle to the application of metal-related materials in biological treatments. While ensuring their therapeutic effect, reducing the in vivo toxicity of metal ions is one of the most important directions for researchers to explore. At this stage, there are mainly two methods; first, by reducing the concentration of metal materials used, and second, by optimizing the metabolism of metal ions. However, the effect is not yet satisfactory. Next, the antiviral mechanism of some metal ions has not yet been explored clearly. Finally, there are many metal nanomaterials, and the current research is limited to a few metal ions. The antiviral properties of other metals still need to be further studied. Based on their excellent properties, the application of metal-based nanomaterials in the antiviral field is a promising research field for expansion.

## Figures and Tables

**Table 1:** Investigations on metal nanomaterials inhibiting viral infections.

Materials	Size/nm	Synthetic Method	Antiviral Mechanism	Detection Methods	Antiviral Types	Ref.
TiO_2_	8	Sonochemistry	Direct inactivating	Hemagglutination assay (HA)	Newcastle virus	[76]
TiO_2_	4–5	Hydrolysis, Calcination		TCID50	H3N2	[26]
TiO_2_	50	Hydrothermal		SEM, Cytopathic effect (CPE), HA	H9N2	[25]
Fe_2_O_3_/Fe_3_O_4_	/	/		Protein conformation analysis	COVID-19, Hepatitis C virus (HCV)	[31]
Ag	10, 75, 110	Commercialization		TCID50	Feline Calicivirus (FCV)	[77]
Cu (II)-zeolite	/	Mechanochemistry		TCID50, HA/NA, RT-PCR, indirect fluorescent antibody (IFA)	H5N1, H5N3	[78]
CuI	100–400	Commercialization		Plaque assay	FCV	[79]
CuI	160	Commercialization		Plaque assay, SDS-PAGE	H1N1	[80]
Cu-doped TiO_2_	20	Sol-gel		qRT-PCR	Human norovirus	[81]
Te/BSA	57 ± 7.6	Redox		TCID50, IFA	Porcine epidemic diarrhea virus	[82]
SnO_2_	50–100	Flame transport synthesis		Plaque assay, fluorescence imaging	Herpes simplex virus-1	[83]
ZnO	100–500	Flame transport synthesis		Reporter Lysis assay	Herpes simplex virus-1	[17]
Ag	10	Commercialization		TCID50, RT-qPCR, IFA	SARS-CoV-2	[84]
Ga	/	/		Plaque assay, fluorescence imaging,	HIV	[85]
PEGylated ZnO-NPs	16–20	Commercialization	Intracellular inhibition (replication)	TCID50	H1N1	[24]
CuO	40	Commercialization		TCID50, IFA	Herpes simplex virus-1	[27]
Fe_3_O_4_	10–15	Hydrothermal		TCID50, RT-PCR	H1N1	[30]
Ga(III) Nanoparticles		Mechanochemistry		TCID50	HIV	[86]
Au@Ag		Seed-mediated growth		TCID50	Coronavirus	[87]
TiO_2_	20–25	/		RT-PCR, TCID50, alkaline single-cell gel electrophoresis (comet)	Noroviruses	[88]
Ag_2_S	3.7, 5.3	Sol-gel	Intracellular inhibition (replication and release)	TCID50	Porcine epidemic diarrhea virus	[35]
CdTe	3.2 ± 0.8	Hydrothermal		TCID50	Pseudorabies virus	[89]
Ag@OTV	2	Sonochemistry	Drug-loading	Caspase-3 activity, TEM	H1N1	[90]
TiO_2_~DNA	~5	Hydrolysis synthesis		TCID50	Influenza A virus	[91]
Ag/TiO_2_	12–32	Sol-gel		/	H1N1, enterovirus	[92]
Au	35	Seed-mediated growth		Immunohistochemistry	Neurotropic virus	[93]

## Data Availability

Data sharing is not applicable for this article.
